# An Important Differential: Extramammary Paget's Disease

**Published:** 2015-03-12

**Authors:** Nicholas Haydon, Francis Ting, James Southwell-Keely

**Affiliations:** Department of Plastic and Reconstructive Surgery, St Vincent's Hospital, Sydney, Australia

**Keywords:** extramammary Paget's disease, perianal cutaneous lesions, skin lesion, scrotal lesion, penile lesion

**Figure:**
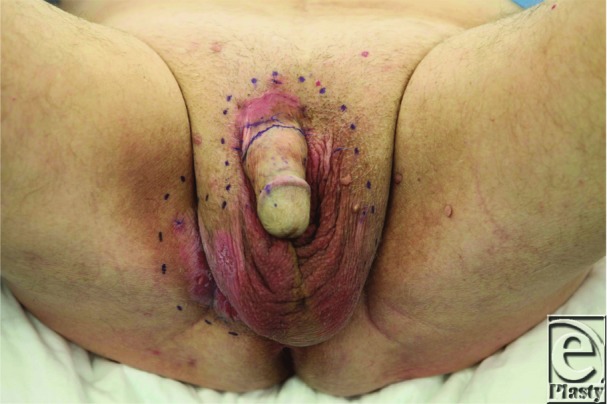

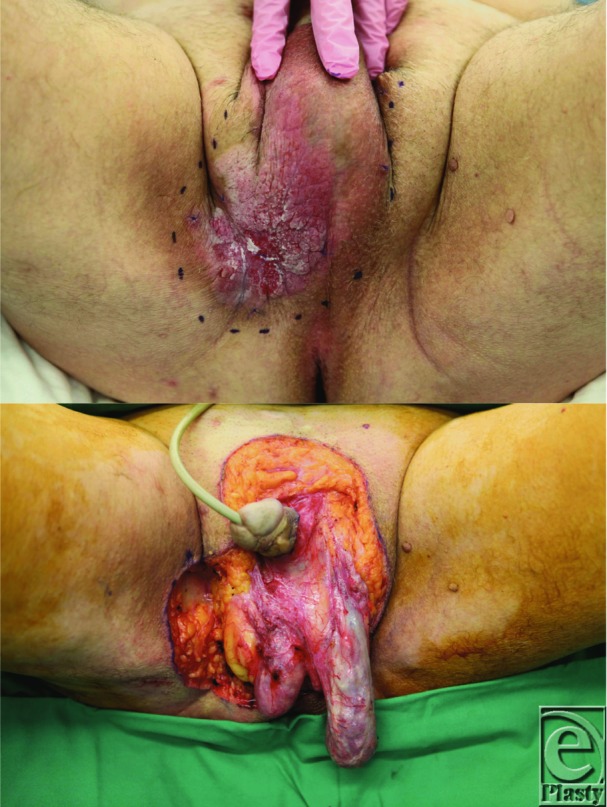


## DESCRIPTION

A 70-year-old man presented with a slow growing painless lesion on his right scrotum and base of penis for 2 years. It caused considerable irritation and discomfort and required wound dressings. He had tried numerous treatments without response.

## QUESTIONS

1. **What are some differential diagnoses for this lesion?**
2. **What is the best way to diagnose this lesion?**
3. **Are other malignancies linked with this lesion?**
4. **What are the surgical and nonsurgical treatment modalities?**

## DISCUSSION

Middle-aged male patients presenting with nonspecific genital or perianal lesions often provide a diagnostic challenge. These lesions are often misdiagnosed as penoscrotal eczema, intertrigo, chronic dermatitis, tinea cruris, candida, or psoriasis. Nonresolution with active treatment should alert physicians for the need for biopsy. Extramammary Paget's disease (EMPD) is a rare disease that is part of a spectrum of intraepithelial adenocarcinomata and accounts for 6.5% of all cutaneous Paget's disease.^1^^,^^2^ Those between the ages of 50 to 80 are more commonly afflicted, with peak age of incidence being 65 years.^2^ Extramammary Paget's disease of the vulva is most common accounting for up to 65% of cases while other areas affected include perianal area, male groin and scrotum, and axillae.^2^

Biopsy and immune-histological testing is the best way to diagnose EMPD. It shares many clinical and histopathological features of Mammary Paget's disease but differs in its pathogenesis and epidemiology.^1^ Histopathological findings include intraepithelial proliferation of large round cells with abundant, pale staining; basophilic cytoplasm; and large, centrally situated nuclei (Paget cells).^2^ Utilization of CK7 and CK20 is useful in the initial evaluation of biopsy specimens, with CK7 having a sensitivity in the range of 86% to 100% for Paget cells in both MPD and EMPD, and CK20 reported to be more specific for EMPD.^2^ To rule out melanoma, S100 and Melan A can be used, and to rule out squamous cell carcinoma in situ, high molecular weight cytokeratin and p63 are of use.^3^

In a review of 197 cases of EMPD, 12% of these patients were found to have an associated concurrent underlying internal malignancy, with the location of the malignancy closely related to the location of the EMPD.^4^ Patients require imaging and procedures as investigation to screen for underlying malignancy. Immunohistochemical staining is useful in identifying Paget's cells. It also helps to delineate primary EMPD, which is not associated with an underlying deep carcinoma, from secondary EMPD, which is associated with an underlying deep carcinoma. The major prognostic factor affecting survival is depth of invasion.^5^^,^^6^ Other factors affecting survival in a series of 76 patients were presence of nodules in the primary tumor, clinically enlarged lymph nodes, elevated serum carcinoembryonic antigen, and lymph node metastases.^5^ Extramammary Paget's disease associated with an underlying visceral malignancy carries a worse prognosis.^4^

Surgical treatment with wide local excision has been the standard of care for EMPD. However, EMPD clinically has poorly delineated margins and microscopic extension of tumor cells can result in recurrence rates that have been reported in the range of 20% to 60%.^7^ “Mapping” biopsies as used in this case are a useful adjunct when planning resection. Moh's micrographic surgery is a technique that allows for complete microscopic margin control through use of horizontal frozen sections of the entire circumference of the excised tumor.^7^ The major methods for reconstruction following wide local excision include split-thickness skin graft, local scrotal flaps, rotary flaps, and primary closure. Anterolateral thigh perforator flaps have also been used with success in one series.^8^ Nonsurgical treatment modalities can be used alone or in conjunction with surgery, and they include radiotherapy, systemic and topical chemotherapy, photodynamic therapy, and laser therapy.^6^

Extramammary Paget's disease is a rare disease with considerable variability in its cutaneous presentation and thus poses a diagnostic challenge to the clinician. Middle-aged male patients with nonspecific penoscrotal lesions not responding to active treatment require early biopsy with the differential of EMPD in mind. Early diagnosis and appropriate referral leads to improved patient outcomes.
